# Effects of Childhood Police Contact on Adolescent Suicidality: A Propensity Score Matched Analysis

**DOI:** 10.1007/s10802-025-01398-8

**Published:** 2026-01-22

**Authors:** Sara J. Schiff, Jocelyn Meza, Steve S. Lee

**Affiliations:** 1 Department of Psychology, University of California Los Angeles, 502 Portola Plaza, Franz Hall, Los Angeles, CA 90095-1563 USA; 2 Department of Psychiatry and Biobehavioral Sciences, David Geffen School of Medicine at UCLA, Los Angeles, CA USA

**Keywords:** Suicide, Juvenile legal system, Preadolescence, Police contact

## Abstract

Youth suicide is increasingly prevalent, is a leading cause of death, and its public health burden is acute. Juvenile Legal System (JLS) involvement is an established correlate of suicidality; however, it is unclear how JLS involvement is nomologically associated with suicidality. Adolescents are situated within ecological contexts (i.e., family, schools, neighborhoods) that likely interact to modify the association of JLS involvement and suicidality. To improve predictive models, rigorous prosecution of this relationship must disentangle related risk/protective factors (i.e., sex/gender, race-ethnicity, discrimination, trauma, familism). Based on 2426 adolescents enrolled in a substudy of the Adolescent Brain and Cognitive Development Study (ABCD), we utilized propensity score matching to test the association of police contact at 10–13 years-old with suicidal outcomes (i.e., self-harm, suicidal ideation, suicide attempt) two years later, covarying for age, education, race-ethnicity, sex/gender, discrimination, adverse childhood events (ACEs), and familism. After adjusting for numerous demographic, experiential, and family-level correlates, police contact did not significantly predict suicidal outcomes two years later. Baseline ACEs positively predicted self-harm and suicidal ideation two years later. Lower familism predicted self-harm, suicidal ideation, and suicide attempts two years later. With inclusion of important risk and protective factors, JLS involvement did not uniquely predict suicidality. Factors closely related to JLS involvement (i.e., ACEs, familism) incremented risk. To address the increasing prevalence of suicidality and the disproportionate impact of suicide on JLS-impacted youth, it is critical to investigate individual and systemic factors, and how they interact, to increase risk for suicidality.

Suicide is the second leading cause of death for youth and young adults 10–24 years old in the U.S. (Centers for Disease Control and Prevention, 2023); recent trends also reveal that its prevalence and public health burden are increasing acutely. For example, suicide in youth (10–14 years old) has increased from 7.23 to 8.75 per 100,000 from 2018 to 2021 (Centers for Disease Control and Prevention, 2021). Rates of death by suicide have also increased significantly by 8.2% annually from 2008 to 2022 in preteens ages 8–12 (Ruch et al., 2024). Approximately 9% of high school students self-reported a suicide attempt during the past year in 2023 (Centers for Disease Control and Prevention, 2024). Critically, history of self-injurious thoughts and behaviors (SITBs), including suicidal ideation, suicide threats/gestures, suicide plan, suicide attempt, and non-suicidal self-injury (NSSI), predicted increased risk for death by suicide among adolescents (Castellví et al., 2017). 50 years of research efforts centered on identifying predictors of youth suicide have yet to systematically consider structural determinants of SITBs, including the impact from involvement in social systems. Without this work, significant innovations in the development and delivery of effective intervention and prevention strategies for youth SITBs are unlikely and the prevalence and burden of youth suicide will remain intractable.

One structural determinant that has established risk for SITBs is the Juvenile Legal System (JLS). Broadly defined, “JLS involvement” encompasses separable points of contact with the legal system; the Sequential Intercept Model (SIM) has identified five key points of diversion to rehabilitative services whereby effective, accessible, and criminologically informed mental health care can be tailored and delivered (Griffin et al., 2015; Yeager et al., 2013). To maximize intervention and prevention effects, these intercepts may also elucidate potential sensitive periods and critical contexts for service delivery. With respect to suicidality and JLS involvement, the SIM was adapted to consist of intercept 0 (prevention), 1 (law enforcement), 2 (courts), 3 (confinement), 4 (re-entry), and 5 (community supervision) (Meza et al., 2022). Relative to the general population, rates of suicide are as much as three times higher in court-involved, non-incarcerated youth and detained youth (Katsman & Jeglic, 2021; Kemp et al., 2016). In addition, incarceration increases risk of death by suicide among youth (Morgan et al., 2022). In terms of past-year prevalence, JLS-impacted youth reported higher rates of suicidal ideation and attempts compared to non-JLS-impacted youth (Stokes et al., 2015). Also, as JLS involvement increased from initial police contact to detention in secure facilities, rates of suicidal ideation and behavior similarly increased (Stokes et al., 2015), providing quasi-experimental evidence that JLS involvement is a potential *causal risk factor* for youth SITBs. Among 12–18 year-old adolescents, suicidal ideation persisted 3 months after initial contact with the JLS (i.e., court appointment) for more than half of youth with previous ideation; this suggests that putative effects on SITBs persist following immediate JLS involvement (Kemp et al., 2021). There is a pressing need to identify unique and shared risk factors for suicidal outcomes across the SIM; however, the prevailing evidence on the associations between JLS involvement and SITB outcomes has focused narrowly on intercept 3, confinement (i.e., detention/incarceration; Stokes et al., 2015). Thus, knowledge about the developmental antecedents and consequences of JLS involvement across intercepts will generate new knowledge about risk-outcome associations as well as resilience-promoting factors.

Given that 84% of police officers report encounters with individuals at risk for suicide, contact with law enforcement – intercept 1 of the SIM – is arguably among the most critical opportunities to identify acute mental health concerns (Meza et al., 2022). For example, suicidal behaviors can be criminalized (i.e., lead to arrest/detainment by law enforcement) or referred for evidence-based mental health care (Meza et al., 2022). Among older adolescents and young adults, interactions with police were positively associated with SITBs (i.e., non-suicidal self-injury, suicidal ideation, suicide attempts) (DeVylder et al., 2017; Jackson et al., 2024). In the U.K., experiences with police stops at age 14 significantly predicted elevated self-harm and attempted suicide at age 17 (Jackson et al., 2021). Even more, among Black adults from the National Survey of American Life, police contact (i.e., unfair stops, searches, questioning, being physically threatened, or abused) was positively associated with negative mental health outcomes including increased suicidal ideation, plans, and attempts (Oh et al., 2017). However, it is unclear if police contact among preadolescents increases risk for SITBs specifically, a critical knowledge gap given this sensitive period of socioemotional development. Preadolescence is a developmental stage marked by rapid changes in emotion regulation, social cognition, and perceptions of authority, during which children become increasingly aware of social stigma and interpersonal threat (Mascia et al., 2023). Because youth in this period have limited autonomy and coping resources relative to older adolescents (Mascia et al., 2023), it is possible that exposure to stressful or adversarial police encounters may be especially disruptive, heightening vulnerability to subsequent SITBs. Indeed, negative police contact predicted more severe depression – a common precursor to SITBs – which is hypothesized to occur through increased stress (Dennison & Finkeldey, 2021; Jackson et al., 2019; Turney, 2021). The present study defined JLS involvement as contact with police officers (i.e., Intercept 1), which will inform effective policing strategies for preadolescents overall and those with mental health needs specifically.

Preadolescents are situated within multiple socioecological contexts (i.e., family, schools, neighborhoods) where factors such as sex/gender, race-ethnicity, discriminatory experiences, trauma history, and social support may modify the association of police contact with engagement in SITBs (Alvarez et al., 2022). The minority stress model contends that myriad social, psychological, and structural factors related to minority status (i.e., sex/gender, racial-ethnic) catalyze stress responses that undermine mental health (Frost & Meyer, 2023). Thus, any putative association between police contact and engagement in SITBs must disentangle other risk (e.g., sexual and gender minority [SGM] status, race-ethnicity, discrimination, adverse childhood events) and protective (e.g., familism) factors that may contribute to both.

JLS-involved youth are at heightened risk for suicide, which is compounded among those with structurally marginalized identities. Structural systems such as racism, sexism, and cisnormativity interact to shape disparities in mental health outcomes and JLS involvement. Ethnoracially minoritized youth are overrepresented at every stage of JLS contact—from arrest to incarceration—and are more likely than their white peers to face punitive outcomes (Fader et al., 2014; Hockenberry & Puzzanchera, 2020; T. Hughes et al., 2020; Robles-Ramamurthy & Watson, 2019; Sickmund et al., 2020). These same youth also face rising suicide rates, with Black and Latinx adolescents experiencing steeper increases in suicide deaths compared to their white peers (Curtin & Hedegaard, 2019; Lindsey et al., 2019), and Hispanic preteens exhibited the greatest increase in suicide deaths from 2001 to 2022 (Ruch et al., 2024). Gender further differentiates risk for sentencing: whereas girls are more likely to be sentenced to group homes than boys, boys are more likely to be sentenced to correctional facilities compared to girls (Tam et al., 2016). However, it is hypothesized that due to expectations on young women to conform to traditional “passive” gender roles, authority figures may be more likely to view young women with aggression criminally compared to similarly behaving young men (Aydt & Corsaro, 2003). In regard to differential risk for suicidal outcomes, girls report more suicidal ideation and attempts (Kokkevi et al., 2012; Miranda-Mendizabal et al., 2019; Nock et al., 2013; Ortin-Peralta et al., 2023; Thompson et al., 2024) whereas boys remain more likely to die by suicide (Miranda-Mendizabal et al., 2019; Sheftall et al., 2016). In addition, a recent scoping review found that JLS-involved adolescent girls exhibited greater SITB risk overall, likely due to their high rates of trauma and mental health needs (Bravo et al., 2025).

Problematically, Black adolescent girls have the highest rates of SITBs overall and demonstrated the sharpest rise in suicide rates over time compared to other gender and racial-ethnic groups (Romanelli et al., 2022). There is also inconsistent evidence that intersectional identities may shape experiences of JLS involvement: White females had a lower probability of pre-adjudication detention than either White or non-White males, but their probability of detention did not differ from non-White females, and White females are more likely than non-White females to receive out of home placements (Guevara et al., 2006). A second study found that White females did not experience differential treatment across the JLS, while African American females tended to receive more lenient outcomes than expected, particularly at intake and petition. African American males showed a mixed pattern—receiving some leniency at intake but facing harsher outcomes at detention and disposition stages (Leiber et al., 2009). However, in a school setting – Intercept 0 (prevention) in the SIM – Black girls were three times more likely than White girls to receive school office referrals—a disparity greater than that between Black and White boys (Morris & Perry, 2017). In addition, Black girls were disproportionately referred for subjective infractions, such as disruptive behavior, dress code violations, and disobedience, which are sensitive to gendered interpretations (Morris & Perry, 2017). Thus, intersectionally, school disciplinary practices may non-randomly penalize African American girls for behaviors seen as violating normative standards of femininity (Morris & Perry, 2017).

Although there is limited research on the effect of policing and suicidal behaviors among transgender youth, in a survey of 600 LGBTQ youth aged 13–24, nearly one-third of transgender and gender-expansive participants reported experiencing police harassment and 60% expressed little to no confidence that police were present to protect them (The Trevor Project, 2020). Further, LGBQ youth collectively reported higher levels of police violence stress than heterosexual youth and depressive symptoms were significantly moderated by LGBQ identity such that police exposure and violence stress compounded to worsen depressive symptoms among the subsample of LGBQ youth (Jackson et al., 2024). Transgender and nonbinary youth also experience significantly higher rates of suicidal ideation and attempts compared to their cisgender peers (Connolly et al., 2016; Eisenberg et al., 2017; McKay et al., 2019; Price-Feeney et al., 2020; Suarez, 2024; Thoma et al., 2019), with gender incongruence (i.e., not feeling aligned with the gender associated with one’s sex assigned at birth) among preadolescents predicting later onset of NSSI and suicidal ideation (Hull et al., 2025). Taken together, youth with multiple structurally marginalized identities are at heightened risk for both suicide and JLS involvement. These findings reaffirm that rigorous empirical models of suicide risk among JLS-involved youth must consider the intersection of race-ethnicity, sex, and gender identity.

Greater exposure to adverse childhood experiences (ACEs) may critically explain or contribute to established associations of elevated suicide risk among structurally marginalized youth with JLS involvement. Defined as stressful or traumatic events occurring in childhood, ACEs disrupt neurodevelopment and impair social, emotional, and cognitive functioning, leading to poor physical and mental health concerns (Boullier & Blair, 2018; Graf et al., 2021). Across geographic regions and types of JLS contact, elevated ACEs increased risk of JLS contact (Graf et al., 2021). In addition, up to 90% of JLS-impacted youth report significant history of ACEs (Baglivio & Epps, 2016; Modrowski et al., 2024). Similarly, greater numbers of ACEs were associated with increased risk of suicidality (Hughes et al., 2017; Sahle et al., 2022). Therefore, understanding the relationship between JLS involvement and youth SITBs requires careful consideration of ACEs. In particular, discrimination – a key component of ACEs – shows strong associations with negative outcomes; high visibility events of police brutality and xenophobia reflect the painful history of persistent structural racism, with nearly 84% of Black and 20% of Latinx youth reporting racial-ethnic police discrimination in the last year, compared to 2.9% of White youth (Zeiders et al., 2021). Discrimination uniquely predicted suicidal ideation above and beyond the effects of depressive symptoms (Madubata et al., 2022). Among pre-adolescents, racial-ethnic discrimination was cross-sectionally associated with increased suicidal ideation and past/current attempts (Argabright et al., 2022) and a recent meta-analysis observed a small, but significant effect of experiencing racial discrimination as a risk factor for suicidal ideation and attempts (Coimbra et al., 2022). Black and Latinx youth are also less likely to be diverted from the JLS to other social services, reflecting biases in clinical decision making (Sickmund et al., 2020). Disruptive behaviors among ethnoracially minoritized adolescents are more criminalized than similar behaviors in White youth (Ramey, 2015). Given these disparities, racial-ethnic discrimination, and ACEs more broadly, must be considered when examining the link between SITBs and police contact.

Aligned with the principles of developmental psychopathology, discontinuity in outcomes likely reveals the intervening influences of moderating and mediating variables. Identifying resilience-promotion factors is essential given that they constitute compelling intervention targets that may alter trajectories of development for ethnoracially diverse youth. Indeed, protective factors are not simply the absence of risk factors; rather protective factors buffer the association between police contact and engagement in SITBs without directly changing the underlying risk factors themselves (Appleby, 1992). Familism, characterized by a cultural emphasis on warmth, interconnectedness, and prioritizing familial obligations over individual needs, mitigated suicide risk predictions among Latinx youth (Campos et al., 2014; Meza & Bath, 2020). Indeed, a review revealed small effect sizes with respect to familism and suicide (Valdivieso-Mora et al., 2016). Family factors are also relevant to suicide among African American communities. Indeed, family support and family cohesion (e.g., emotional connectedness within a family) were inversely correlated with suicidal ideation among African American college students (Harris & Molock, 2000). Among African American adolescents, increased family support was associated with decreased suicide ideation and attempts (Matlin et al., 2011). Even more, a recent review highlighted that family support constructs (e.g., parental emotional support, closeness, parental monitoring) consistently buffered predictions of mental health more broadly from racial discrimination among Black youth (Neblett, 2023). Family support is also a particularly relevant construct to JLS-involved youth (Snyder et al., 2024) given that family-based interventions (e.g., multisystemic therapy) significantly reduce recidivism in youth (Aalsma et al., 2015). We contend that family-related protective factors (i.e., familism) must also be accounted for in discerning if/how police contact is associated with youth SITBs.

Overall, JLS involvement and engagement in SITBs are associated with highly consequential outcomes for youth. However, given that they are embedded in a broad network of multiple risk and protective factors, putative JLS involvement effects on SITBs must be disentangled from these other intervening factors. In addition, most of the preexistent literature focuses on adolescent populations and points of JLS contact later in the SIM (i.e., detainment), highlighting the need for studies focused on preadolescence and earlier points of JLS involvement (i.e., police contact prior to age 10). Employing a prospective longitudinal study of 2426 racially and ethnically diverse adolescents, we used propensity score matching to test the longitudinal association of baseline police contact (i.e., ever been stopped/questioned by police) with SITB outcomes (i.e., self-harm, suicide ideation, suicide attempts) at a 2-year follow-up in a substudy of the Adolescent Brain and Cognitive Development (ABCD) study (i.e., a nationally representative sample of U.S. youth). The putative effect(s) of police contact covaried for age, education, race-ethnicity, sex/gender, experiences of discrimination, ACEs, and familism. Propensity scores were calculated matching on the aforementioned covariates, along with potentially confounding measures regarding parent/family factors, community cohesion, peer relationships, school engagement, psychiatric concerns, positive life events, and impulsivity. Baseline police contact (i.e., ever been stopped/questioned by police) and suicide attempts (i.e., lifetime history of self-harm, suicidal ideation, and suicide attempt) at the follow-up were estimated dichotomously. Using propensity score matching techniques, we hypothesized that police contact at baseline would positively predict SITBs at follow-up, even accounting for confounding factors.

## Methods

### Participants and Procedures

The current study utilized data from the Adolescent Brain and Cognitive Development (ABCD) study, the largest longitudinal study of brain development and child health in the U.S. At ABCD study baseline, 11,875 children ages 8–11 years old were recruited to be nationally representative with respect to sex, race, ethnicity, urban/rural residency, and socioeconomic status. Data are currently available for the baseline and 1-, 2-, 3-, and 4-year follow-up waves. This study primarily used data from participants at the 2-year and 4-year follow up assessments (ages 10–13 and 12–15 years, respectively), as these waves developmentally reflected sensitive periods with respect to engagement in SITBs and contained relevant measures to our study aims. A substudy focused on the social development of participants was offered to individuals at either their 1- or 2-year follow-up assessment and included items assessing for police contact. Participants were assessed in-person once a year, with additional assessments for the Social Development substudy occurring either at or between regular study visits. For the purposes of the present study, the 2-year follow-up and the Social Development assessment of the ABCD study will be referred to as “baseline” and the 4-year follow-up of the ABCD study will be referred to as “follow-up.” Data used for this study included only individuals who have completed the Social Development substudy and thus have available police contact data (*N* = 2426). Self- and parent-report measures were gathered to assess demographics (i.e., race-ethnicity, sex/gender, age, grade level), SITB outcomes (i.e., lifetime history of self harm, suicidal ideation, and suicide attempt), legal system involvement (i.e., police contact), discrimination, ACEs, and familism. Preliminary findings revealed expected variance with respect to constructs of interest considering the sample is comprised of preadolescents who are not designated “high risk” (see below for additional details).

### Measures

#### Self-Injurious Thoughts and Behaviors

##### Kiddie Schedule for Affective Disorders and Schizophrenia (KSADS) (Townsend et al., 2020)

The Kiddie Schedule for Affective Disorder and Schizophrenia (KSADS) for Diagnostic and Statistical Manual for Mental Disorders (DSM-5) suicide module was administered to youth and parents at the baseline and follow-up waves of data collection (Townsend et al., 2020). This module assessed for past and current passive and active (e.g., non-specific, method, intent, plan) thoughts of suicide, interrupted and aborted suicide attempts, and NSSI. Parent and youth report on these items were combined such that the behavior was endorsed for a participant if either parent or youth provided a positive endorsement (De Los Reyes et al., 2015; Kuhn et al., 2017). These variables were then combined to create two items reflecting lifetime history of self-harm and lifetime history of suicidal ideation. Specifically, participants were designated as endorsing self-harm (i.e., 1 = present, 0 = absent) if they endorsed any of the following behaviors in the past or currently: interrupted suicide attempt, aborted suicide attempt, or NSSI. Participants were designated as endorsing suicidal ideation (i.e., 1 = present, 0 = absent) if they endorsed any of the following types of suicidal ideation in the past or currently: passive, active non-specific, active method defined, active intent established, active plan developed. Suicide attempt was estimated from combined reports from parent and child (i.e., 1 = history of suicide attempt, 0 = no history of suicide attempt). 4.7% of the sample endorsed lifetime history of self-harm and 6.8% of the sample at follow-up endorsed lifetime history of SI. 1.2% of the sample endorsed ever making a suicide attempt.

#### Juvenile Legal System Involvement

##### Police Contact

ABCD assessed JLS involvement (i.e., police contact) in the Social Development substudy using the Social Development Child Reported Delinquency Scale, administered at either the 1- or 2-year follow up waves of ABCD data collection (i.e., “baseline” for the present study). Youth and parents were separately asked to report on the youth’s past contact with police with the following questions: “Have you [your child] been questioned and stopped by the police?” and “Have you [your child] been arrested?” and response options included: Never, Once or twice, More often, I don’t know, and I’d rather not say. Given base rates for each response category were too small to compare empirically, answer choices were dichotomized. Data were then combined using the “or” rule if endorsed by the youth or parent with respect to ever having police contact or being arrested, similarly due to low base rates across youth and parent report. 5.1% of the sample endorsed a lifetime history of having ever been questioned/stopped by the police and 0.4% having ever been arrested. Finally, considering being arrested necessarily includes being “stopped and questioned by the police,” a single dichotomous police contact item was created (5.2% of the sample endorsed at least one of these items).

#### Demographics

ABCD youth self-identified race across White, Black/African American, American Indian/Native American, Alaska Native, Native Hawaiian, Guamanian, Samoan, Other Pacific Islander, Asian Indian, Chinese, Filipino, Japanese, Korean, Vietnamese, Other Asian, and Other Race categories. Participants were also asked to indicate whether they identify as Hispanic/Latino/Latina. These racial-ethnic categories were then collapsed by the ABCD study to form the following groups: Black, Hispanic/Latinx, White, Asian, and Other. At baseline, 28.6% of the sample identified as Black (*n* = 694), 10.7% identified as Hispanic/Latinx (*n* = 260), 47.9% identified as White (*n* = 1161), 1.6% identified as Asian (*n* = 38), and 11.3% identified as another racial/ethnic category, including biracial or multiracial (*n* = 273). 50.2% of the sample self-reported their gender as male, 45.4% as female, and 0.5% as a gender minority (i.e., trans male, trans female, genderqueer, other). Participant grade was also measured, with the majority of participants being in 6th grade (44.1%) at baseline.

#### Adverse Childhood Experiences (ACEs)

##### Life Events Scale (Grant et al., 2004; Hoffman et al., 2019; Tiet et al., 1998)

Adverse childhood experiences (ACEs) were measured at baseline with a 26-item measure. Participants reported whether they experienced negative life events such as “Someone in family died” and “Was a victim of crime/violence/assault,” and “Family member had a mental or emotional problem.” A sum of the total number of negative life events participants experienced (higher scores reflecting more ACEs) was created and used in the present analyses.

#### Discrimination

##### Measure of Perceived Discrimination (Phinney et al., 1998)

Youth self-reported 7 items with respect to their experiences of discrimination at baseline on an adapted version of the “Measure of Perceived Discrimination,” which assesses frequency of unfair or negative treatment due to ethnic background and was developed by the ABCD study. Items included “How often do the following people treat you unfairly or negatively because of your ethnic background? [Teachers, Other adults outside of school, Other students],” “I feel that others behave in an unfair or negative way towards my ethnic group,” and “I don’t feel accepted by other Americans.” Items were scored on a 5-point Likert scale indicating the frequency that these events occurred (1 = Almost never, 2 = Rarely, 3 = Sometimes, 4 = Often, 5 = Very often). Scores were averaged across all items with higher scores indicating a greater degree of perceived unfair treatment. The published reliability of this scale was 0.81 (Phinney et al., 1998).

#### Familism

##### Youth Mexican American Cultural Values Scale (Knight et al., 2010)

Familism was assessed at baseline using the self-reported Youth Mexican American Cultural Values Scale which was administered to all participants regardless of their cultural background (Knight et al., 2010). Used previously in racially-ethnically heterogenous samples (Finlay et al., 2014; Smith et al., 2017), items were scored on a 5-point Likert scale indicating the extent to which statements are true of their family (1 = Not at all, 2 = A little, 3 = Somewhat, 4 = Very much, 5 = Completely). We calculated a “familism-support” summary score by averaging all familism/family support-related items of the measure (Mean = 4.14; range = 1–5). As previously reported in a study of the psychometric properties of the various subscales of this measure, internal consistency coefficients (i.e., Cronbach’s alphas) for adolescent report on “familism-support” was 0.67 (Knight et al., 2010). Sample items from the “familism-support” subscale include: “Parents should teach their children that the family will always come first,” and “Family provides a sense of security because they will always be there for you.”

#### Pretreatment Covariates Included in Propensity Score Matching

All pretreatment variables (e.g., demographic variables, baseline risk factors, clinical history) conceptualized to relate to both treatment and outcomes were included in the model and used to generate propensity scores. Covariates included the variables above (i.e., age, grade, sex/gender, race-ethnicity, ACEs, discrimination, and familism) as well as parent and family-related variables (i.e., parent education, parent employment status, family income, family economic stress, family conflict, family cohesion), community-related variables (i.e., community cohesion, neighborhood safety and crime), peer relationships (i.e., involvement with prosocial and rule breaking/delinquent peers, affiliation with “protective” peers, peer aggression perpetration and victimization), schooling concerns (i.e., school disengagement, detention/suspension history), psychiatric concerns (i.e., depression, anxiety, ADHD, oppositional defiance, conduct problems), positive life events (i.e., life events, such as got a new brother/sister, rated as a “good experience”), temperament (i.e., behavioral inhibition, negative urgency, sensation seeking), and pubertal development.

### Data Analytic Plan

The present prospective study used data from the 2-year and 4-year follow-ups of the ABCD study (i.e., “baseline” and “follow-up,” respectively). All data were evaluated for standard statistical assumptions (i.e., normality, skewness, kurtosis) prior to analyses and alternative models (e.g., log-based) were considered to assess appropriate model specification. Point-biserial correlations were run to assess the associations between our police contact variable and relevant covariates (i.e., ACEs, discrimination, and familism) at baseline, prior to propensity score matching.

We used the Twang package in R to conduct propensity score matching to investigate the prediction of dichotomously-defined suicidal outcomes (i.e., lifetime history of self-harm, suicidal ideation, and/or suicide attempt) from dichotomous police contact (Griffin et al., 2014). A propensity score is the probability that an individual has received a specific exposure (i.e., police contact) given a set of observed covariates. Propensity scores help minimize bias in estimating treatment effects by balancing exposure groups based on the observed pretreatment covariates included in the propensity score model, thus removing potential confounding biases from observational studies. By summarizing all potential (measured) confounders into a single propensity score, the process of controlling for confounding bias when estimating exposure effects is simplified. Thus, the propensity score weights the groups (i.e., police contact vs. no police contact) so that they are balanced on all of the observed pretreatment covariates (i.e., variables that differ between individuals in the exposure groups and are measured before they receive the assigned exposure) that go into the propensity score model (McCaffrey et al., 2004). In other words, propensity score matching compared individuals with past police contact – versus those without past police contact – while simultaneously matching the two groups on theoretically- and empirically-driven confounders of this relationship.

This approach assumes that (a) conditional on the included covariates, treatment assignment is independent of potential outcomes (unconfoundedness); (b) there is sufficient overlap in propensity scores between groups (common support); (c) the propensity score model is correctly specified and includes all relevant covariates; and (d) each participant’s potential outcome is independent of other participants’ treatment assignments. Following matching, we evaluated covariate balance using standardized mean differences and graphical diagnostics to ensure these assumptions are reasonably met before estimating treatment effects. Specifically, the Twang package algorithm iteratively fits boosted regression trees to optimize covariate balance between treated and comparison groups. Covariate balance was then assessed using standardized mean differences, ensuring that all pretreatment variables achieved acceptable balance after weighting. Then, outcome analyses were conducted on the weighted sample, which reduces bias attributable to confounding and strengthens causal inference compared to unadjusted models.

In the present study, we created propensity scores balanced on the identified pretreatment covariates above using gradient boosted models. We employed the Average Treatment Effect (ATE) on the population, rather than the Average Treatment Effect on the Treated (ATT) given our interest in understanding if police contact prospectively predicts suicidal outcomes (see Greifer & Stuart (2021) for additional details on selecting target estimates). For missing values in the covariates, the Twang package constructs weights that balance rates of missingness across the police contact vs. no police contact groups, thereby mitigating potential bias due to missingness (Ridgeway et al., 2021).

Three sets of logistic regression analyses evaluated whether police contact was associated with increased risk for our three suicidal outcomes (i.e., dichotomous lifetime history of self-harm, suicidal ideation, and suicide attempts), covarying for age, grade, sex/gender, race-ethnicity, ACEs, discrimination, and familism.

## Results

See Table 1 for descriptive statistics of demographics and key baseline variables. Point-biserial correlations revealed that police contact at baseline was significantly associated with baseline experiences of discrimination (*r* = 0.07, *p* = 0.001) and ACEs (*r* = 0.11, *p* < 0.001), but not baseline familism (*r* = 0.02, *p* = 0.46). See Table 2 for full correlations matrix.

**Table 1:** Descriptive statistics of demographics and key variables at baseline

	M (SD), Range or % of Sample
Age	11.52 (0.72), 9.0–14.0
Race*	
Black	28.6
White	47.9
Hispanic/Latinx	10.7
Asian	1.6
Other	11.3
Gender*	
Male	50.2
Female	45.4
Gender minority (i.e., trans male, trans female, genderqueer, other)	0.5
Grade*	
3rd Grade	0.1
4th Grade	0.9
5th Grade	12.4
6th Grade	44.1
7th Grade	32.6
8th Grade	6.1
9th Grade	0.0
Police Contact	5.2
Adverse Childhood Events	2.58 (2.32); 0–16
Discrimination	1.18 (0.41); 1–5
Familism	4.12 (0.67); 1–5
Suicidal Outcomes	
Ever Self Harm	4.7
Ever Suicidal Ideation	6.8
Ever Suicide Attempt	1.2

*Totals do not add up to 100 due to missing data

**Table 2:** Correlations matrix of baseline variables

	1	2	3	4	5	6	7	8
1. Age	–							
2. Gender	−0.03	–						
3. Education	0.72**	0.02	–					
4. Race-Ethnicity	0.01	0.03	0.01	–				
5. Police Contact	0.06**	−0.095**	0.04	0.06**	–			
6. Adverse Childhood Events	−0.03	−0.02	−0.05*	0.01	0.11**	–		
7. Discrimination	0.05*	−0.02	0.02	0.14**	0.07**	0.18**	–	
8. Familism	−0.11**	−0.07**	−0.14**	0.05*	0.02	0.01	−0.01	–

** Correlation is significant at the 0.01 level; * Correlation is significant at the 0.05 level

Prior to propensity score matching, 25 out of 40 pretreatment covariates were significantly imbalanced (i.e., differed significantly; *p* < 0.05) across exposure groups (police contact vs. no police contact). After employing the es.mean stopping criteria (i.e., algorithm determines the iteration that minimizes the average absolute standard effect size), only 5 out of 40 pretreatment covariates remained imbalanced (i.e., participant age, participant grade, primary parent education, family income, and parent-reported positive life events), suggesting propensity score estimation improved the balance in potential confounding variables for nearly all covariates included in the model. Because unbalanced covariates may continue to confound treatment effect estimates, we included these variables as covariates in predictive models and interpret findings cautiously.

The overall model predicting lifetime history of self-harm was statistically significant, Wald χ^2^(11) = 3.79, *p* < 0.001, indicating that the predictors jointly contributed to explaining self-harm history. The model accounted for approximately 23% of the variance in the outcome (pseudo *R*^2^ = 0.23). The overall model predicting lifetime history of suicidal ideation was statistically significant, Wald χ^2^(11) = 2.65, *p* = 0.002, indicating that the predictors jointly contributed to explaining suicidal ideation history. This model accounted for approximately 9% of the variance in the outcome (pseudo *R*^2^ = 0.09). The overall model predicting lifetime history of suicide attempts was statistically significant, Wald χ^2^(11) = 2.33, *p* = 0.008, indicating that the predictors jointly contributed to explaining suicide attempt history. This model accounted for approximately 31% of the variance in the outcome (pseudo *R*^2^ = 0.31).

Adjusted propensity-score weighted regression models revealed that baseline police contact did not significantly predict any SITB outcomes (i.e., dichotomous lifetime history of self-harm, suicidal ideation, or suicide attempts) at follow-up. However, key covariates significantly predicted SITB outcomes. Gender identity (*b* = 0.17, *SE* = 0.04*, p* < 0.001), ACEs (*b* = 0.03, *SE* = 0.01*, p* = 0.02), and familism (*b* = −0.08, *SE* = 0.03*, p* = 0.01) positively predicted lifetime history of self-harm. In addition, ACEs positively predicted (*b* = 0.03, *SE* = 0.01*, p* = 0.049) and familism inversely predicted (*b* = −0.13, *SE* = 0.04*, p* = 0.005) lifetime history of suicidal ideation. Last, familism inversely predicted lifetime history of suicide attempts (*b* = −0.03, *SE* = 0.02*, p* = 0.03). See Table 3 for full results.

**Table 3:** Propensity score matched associations of police contact and covariates with suicidal outcomes

Dependent variable	Predictors	*b*	SE	*p*
Lifetime History of Self Harm	Police contact	0.02	0.06	0.69
	Age	0.02	0.05	0.60
	Education	−0.05	0.04	0.20
	Gender	0.16	0.04	< 0.001*
	Race-Ethnicity	0.03	0.02	0.29
	Adverse childhood events	0.02	0.01	0.03*
	Discrimination	0.02	0.06	0.80
	Familism	−0.08	0.03	0.01*
Lifetime History of Suicidal Ideation	Police contact	0.02	0.08	0.76
	Age	−0.01	0.04	0.73
	Education	−0.02	0.04	0.67
	Gender	0.05	0.05	0.35
	Race-Ethnicity	0.02	0.02	0.32
	Adverse childhood events	0.02	0.01	0.04*
	Discrimination	−0.03	0.06	0.58
	Familism	−0.11	0.04	0.01*
Lifetime History of Suicide Attempts	Police contact	0.06	0.05	0.27
	Age	−0.01	0.02	0.72
	Education	−0.03	0.04	0.40
	Gender	0.06	0.04	0.10
	Race-Ethnicity	0.02	0.02	0.43
	Adverse childhood events	0.01	0.01	0.14
	Discrimination	0.02	0.06	0.70
	Familism	−0.03	0.01	0.03*

*Association is significant at the 0.05 level

## Discussion

This study utilized propensity score matching to examine if baseline police contact – an early indicator of JLS involvement and an established risk factor for SITBs – predicted SITBs (i.e., lifetime history of self-harm, suicidal ideation, and suicide attempts) two years later. Compared to traditional regression-based covariate adjustment, propensity score matching offers a stronger approach for reducing selection bias in observational research to make causal inferences. By creating groups of participants who are balanced on measured covariates prior to estimating treatment effects, propensity score matching more closely approximates the conditions of a randomized experiment. This design-based strategy enhances the internal validity of findings by reducing reliance on model specification and assumptions inherent in regression adjustment, which may inadequately account for systematic differences between groups. The associations found in this study were thus robust to a wide range of possible confounders spanning ACEs, discrimination, familism, and other demographic covariates (i.e., age, grade, sex/gender, race-ethnicity). With adjustment for these possible confounders, findings did not support a potential causal role for police contact with respect to SITBs two years later. However, police contact was significantly associated with key baseline covariates (i.e., discrimination, ACEs) and baseline ACEs significantly predicted suicidal outcomes.

These results both converge with and diverge from literature on risk and protective factors for youth SITBs. Broadly, JLS involvement – and police contact more specifically – is an established risk factor for suicidality. However, this evidence is based almost entirely on adolescents or adults with few, if any, focused on pre-adolescents. While police contact in preadolescence did not predict SITBs two years later, this likely reflects relatively low base rates of police contact at this age. Indeed, some U.S. states prohibit detention of youth younger than age 12 by police (Development Services Group, Inc., 2024); this would significantly delimit the association of negative police contact with different constructs. In the current study, 5.2% of youth in the nationally representative ABCD sample had police contact. A complementary strategy to improve traction on the association of police contact among preadolescents and suicide risk is use of high-risk samples (e.g., child welfare involvement) that are likely to yield more police contact. For example, in the New South Wales Child Development Study, contact with child protection services positively predicted any police contact by age 13 years (i.e., as a person of interest, victim, or witness); in fact, among the approximately 14,000 children with any police contact, about half had prior contact with the child protection system (Athanassiou et al., 2024). Thus, future studies should examine this association among populations with a higher density of risk, which may include earlier and more extensive police contact along the SIM. However, higher-risk samples are challenged by the relative density of risk/confounding factors, thus threatening strong inferences. Nevertheless, if the association of JLS contact and suicidality is more prevalent in higher-risk populations, early prevention efforts may support these vulnerable populations. For example, socio-emotional learning programs (e.g., Strong Kids, PATHS, Good Behavior Game) effectively mitigated suicide risk among preadolescents within school settings (Yepez-Coello et al., 2025). A primary prevention, school-based framework is well-positioned to significantly reduce the occurrence of suicidality amongst these acutely at-risk populations.

Even so, early police contact is one of the strongest predictors of arrest, as well as detention/incarceration later in life, particularly for ethnoracially minoritized populations (Crutchfield et al., 2009; Liberman et al., 2014; Mcara & Mcvie, 2005; McGlynn-Wright et al., 2022; Wiley & Esbensen, 2016). We contend that additional, prospective follow ups across developmental periods are needed to investigate the association between police contact and SITBs, through the potential mediator of arrest or detention/incarceration. Thus, further longitudinal studies must be conducted to assess for the long-term impact of early exposure to police contact in preadolescence.

Although police contact did not directly predict later SITBs, ACEs did show independent prediction. One possible hypothesis is that experiences of ACEs may not only precede but also intensify and accumulate as a consequence of early police contact, reflecting a cascading process in which police contact can lead to further ACEs (i.e., parental incarceration and trauma) and this accumulation of ACEs may, in turn, exacerbate SITB risk. Future research is needed to further test this hypothesis, although there is preliminary evidence to support this (Geller, 2021; Kemp & Sheerin, 2022; Testa et al., 2022; Wiley & Esbensen, 2016). How the construct of police contact is specifically operationalized (i.e., have you ever been stopped/questioned by the police) may also narrowly and insufficiently reflect the phenomenology of JLS involvement. For example, indirect experiences with police (i.e., parental legal system involvement and parental incarceration) also predict increased risk for SITBs (Bravo et al., 2024; Schiff et al., 2024). In addition, the cumulative effect of police contact with additional traumatic experiences may increase risk for engagement in SITBs (rather than police contact alone): for example, adolescents with four or more ACEs reported more suicidal thoughts compared to those without ACEs (Suh et al., 2024), supporting the notion that when individuals experience multiple, potentially compounding, ACEs, they have a higher risk for engagement in SITBs. Therefore, future research must consider both a “unified” approach to ACEs (e.g., composite of multiple indicators) as well as a more “diversified” approach that differentially considers indicators of ACEs, including the potential for non-linear associations. If data suggest different types or severities of suicidal outcomes dependent on the number or nature of ACEs, early detection and screening efforts should include assessments of ACEs to prevent future suicidal outcomes.

In line with previous research, familism inversely predicted SITBs, suggesting familism is a promising resilience-promoting factor. Of note, the present study focused specifically on familism, which is particularly relevant to Latinx communities. However, familism inversely predicted SITBs in the entire sample. Future work should investigate whether familism has a stronger effect on SITBs among specific racial-ethnic subgroups versus others. These results also highlight that intervention-induced strengthening of familism may protect against engagement in SITBs for preadolescents. Thus, interventions and treatment modalities that capitalize on family relationships as protective factors (e.g., behavioral therapies, attachment-based therapies) may effectively mitigate risk for SITBs (Sullivan et al., 2023), particularly among JLS involved youth (Snyder et al., 2024). Indeed, multiple interventions that target the family support system – including the Family Intervention for Suicide Prevention – are rooted in cognitive-behavioral and family systems theory and are designed to activate family support and increase problem solving strategies (Andriessen & Krysinska, 2016; Asarnow et al., 2009). In addition, preadolescence signifies a critical period where children experience significant changes in physical, hormonal, cognitive, behavioral, and emotional development, and thus it represents a crucial context where protective interventions (e.g., parental support) can be implemented to better support youth through this period of change and promote positive mental well-being. Thus, continued research must be conducted to develop family-based interventions specifically targeting this period of development.

### Limitations

The present study utilized innovative propensity score matching techniques to assess the isolated impact of police contact on suicidal outcomes using a large nationally representative sample of preadolescents. However, key limitations to this study preclude definitive conclusions about the association between these constructs. First, police contact was assessed in a way that may equally weight a diverse range of police contact experiences (i.e., positive and negative) that are differentially associated with risk for SITBs. In addition, the ABCD study did not measure involvement in the JLS across additional intercepts of the SIM, precluding inferences about the putative effects of more substantial JLS involvement (i.e., court involvement, incarceration) on suicidal outcomes. However, the present sample consisted of 10–13-year-old youth at baseline, which reflects a developmental time period where JLS involvement is likely to be more circumscribed, at least in an unselected sample. In that sense, the estimate of JLS involvement is likely developmentally appropriate.

### Future Directions

To address the increasing prevalence of youth suicidal outcomes and the disproportionate burden of suicide on ethnoracially minoritized individuals, it is critical to investigate both the individual and systemic factors that increase risk for engagement in SITBs. In particular, identifying the unique and potentially compounded effect(s) of key risk and protective factors – spanning JLS involvement, gender and racial-ethnic identity, ACEs, discrimination, and familism – with accounting for a number of potential confounding factors, is an important innovation. We await additional rigorous tests of these associations across more diverse designs, participant characteristics, etc. that are expected to accelerate innovations in intervention and prevention development. These innovations are necessary to reduce the significant clinical and public health burden associated with suicidality in youth.

## Data Availability

Data used in the preparation of this article were obtained from the Adolescent Brain Cognitive Development^SM^ (ABCD) Study (https://abcdstudy.org), held in the NIMH Data Archive (NDA). The ABCD Study® is supported by the National Institutes of Health and additional federal partners under award numbers U01DA041048, U01DA050989, U01DA051016, U01DA041022, U01DA051018, U01DA051037, U01DA050987, U01DA041174, U01DA041106, U01DA041117, U01DA041028, U01DA041134, U01DA050988, U01DA051039, U01DA041156, U01DA041025, U01DA041120, U01DA051038, U01DA041148, U01DA041093, U01DA041089, U24DA041123, U24DA041147. A full list of supporters is available at https://abcdstudy.org/federal-partners.html. ABCD consortium investigators designed and implemented the study and/or provided data but did not necessarily participate in the analysis or writing of this report. This manuscript reflects the views of the authors and may not reflect the opinions or views of the NIH or ABCD consortium investigators. The ABCD data repository grows and changes over time. The ABCD data used in this report came from the 2024 Data Release 5.1 (DOI: 10.15154/z563-zd24).
